# Effect evaluation of a two-year complex intervention to reduce loneliness in non-institutionalised elderly Dutch people

**DOI:** 10.1186/1471-2458-13-984

**Published:** 2013-10-21

**Authors:** Rianne Honigh-de Vlaming, Annemien Haveman-Nies, Judith Heinrich, Pieter van‵t Veer, Lisette CPGM de Groot

**Affiliations:** 1 Wageningen University, Division of Human Nutrition, Academic Collaborative Centre AGORA, P.O. Box 8129, 6700, Wageningen, EV, the Netherlands; 2 GGD Noord-en Oost-Gelderland (Community Health Service), Academic Collaborative Centre AGORA, P.O. Box 51, 7300, Apeldoorn, AB, the Netherlands

**Keywords:** Evaluation, Loneliness, Public health, Healthy ageing, Complex intervention, Elderly people

## Abstract

**Background:**

Public health policy calls for intervention programmes to reduce loneliness in the ageing population. So far, numerous loneliness interventions have been developed, with effectiveness demonstrated for few of these interventions. The loneliness intervention described in this manuscript distinguishes itself from others by including multiple intervention components and targeting individuals and their environment. Intervention components included a mass media campaign, information meetings, psychosocial group courses, social activities organised by neighbours, and training of intermediaries. The aim of this manuscript is to study the effects of this integrated approach on initial and long-term outcomes.

**Methods:**

A quasi-experimental pre-test post-test intervention study was conducted among non-institutionalised elderly people aged 65 years and over to evaluate the effectiveness of the intervention by comparing the intervention community and the control community. Data on outputs, initial and long-term outcomes, and the overall goal were collected by self-administered questionnaires. Data of 858 elderly people were available for the analyses. To assess the effect linear regression analyses with adjustments for age, gender, church attendance, and mental health were used. In addition, the process evaluation provided information about the reach of the intervention components.

**Results:**

After two years, 39% of the elderly people were familiar with the intervention programme. The intervention group scored more favourably than the control group on three subscales of the initial outcome, motivation (−4.4%, 95% CI−8.3-−0.7), perceived social support (−8.2%, 95% CI−13.6-−2.4), and subjective norm (−11.5%, 95% CI−17.4-−5.4). However, no overall effects were observed for the long-term outcome, social support, and overall goal, loneliness.

**Conclusions:**

Two years after its initiation the reach of the intervention programme was modest. Though no effect of the complex intervention was found on social support and loneliness, more favourable scores on loneliness literacy subscales were induced.

## Background

### Problem definition

Loneliness among elderly people is of growing public concern despite the fact that loneliness does not increase by age itself. Only the oldest-old are often found to be the most lonely [1-6]. However, prevalence estimates of loneliness among non-institutionalised elderly people aged 65 years and over are substantial, ranging from 28% to 63% [7-9]. In addition, age-related live-events, such as retirement, moving to sheltered housing, death of a partner or other relatives, and age-related health problems, affect, on the one hand, the social network ties and, on the other hand, the social support needs of elderly people–two important factors related to loneliness [3-5,10-13]. Furthermore, family structures are changing, i.e. decreasing number of off-spring and increasing distances between family members due to migration [14-16], and new policies emphasise independence, individual responsibility, and societal participation of citizens in old age [17-19]. As the absolute number of older people in general and of the oldest-old is increasing [11,20,21], loneliness prevention for this age group is more than legitimised.

Loneliness has often been defined as the unpleasant or inadmissible lack of the (quality of) certain relationships [10,22]. Loneliness can be reduced by either improvement of network quality or coping with feelings of loneliness [23-25]. Numerous loneliness interventions have been developed during the last decades for very different target groups (general population, high-risk groups, or intermediaries) using different approaches (individual, group, and social environment interventions) [23,26-29]. To date, evidence about the effectiveness of loneliness interventions has been limited because intervention studies use weak research designs, do not use a valid indicator for measuring loneliness, focus only on short-term outcomes, or lack process indicators to gain better understanding about the achievement of the desired outcomes [26-28]. In the Netherlands, 18 loneliness interventions were, almost uniformly, evaluated with an experimental study design. It appeared that loneliness was significantly reduced in two of these interventions: an individual at-home intervention for elderly persons with a chronic disease and a group intervention in a residential care home including discussion groups and coffee breaks. Limited insight into the causes of loneliness in the target population, a one-sided focus on network development, difficulties in reaching the target group, and approaching a too wide target group were identified as reasons for ineffectiveness of the other interventions [23,30]. Furthermore, international reviews have shown that, of the interventions accompanied by (high-quality) effect evaluations, only a limited number have proved to reduce feelings of loneliness. Most promising are group interventions involving an educational component and social activities targeting specific groups of people. Further, involvement of the target population in the planning, development, and delivery of activities, and the utilisation of existing community resources, have been shown to facilitate the development of effective interventions [26,27].

### Development of *Healthy Ageing* intervention

Notwithstanding the limited availability of evidence-based loneliness interventions, public health policy calls for intervention programmes for the prevention of loneliness and the stimulation of social engagement on the community level [17-19,31]. Accordingly, the municipality of Epe, a rural municipality in the eastern part of the Netherlands, decided to start with a community intervention for the prevention of loneliness among non-institutionalised elderly people, called *Healthy Ageing*. A local project group was established, consisting of the regional mental health service, the regional community health service, the local elderly welfare organisation, and the municipality. This project group was mainly responsible for the development of the intervention.

In the development phase, a qualitative context analysis and a quantitative needs assessment were executed in addition to a careful review of the literature. Groups at high risk of loneliness were identified by secondary analyses of the *Elderly Health Survey 2005* of the community health service [7], e.g. elderly persons with physical limitations, a low income, and mild mental disabilities, and recently widowed persons. Further, interviews with elderly people, professionals, and policymakers gave insights into the needs and opportunities for promoting healthy ageing [32]. Staying independent and remaining socially engaged appeared to be important values for elderly people. Furthermore, elderly people preferred to focus on abilities instead of disabilities. Therefore, the project group decided for a positive approach for health promotion. As a result, ‘*Healthy Ageing’* was chosen as project name. Furthermore, both qualitative and quantitative research showed the importance of offering services for elderly in the neighbourhood and the special meaning of neighbours [32,33]. In addition, strategies to reduce loneliness were derived from Van Tilburg (1988) who identified three potential pathways to reduce feelings of loneliness, namely network development, lowering of standards and adjusting the relevance of the experience loneliness [25]. These pathways were combined in the project with a focus on network development. Furthermore, the project group members brought their own expertise in the field of health promotion, health communication, social-recreational activities and preventive psychology. Finally, based on experiences in other community interventions [34-36], an integrated approach was initiated, combining multiple strategies; delivering intervention components to different target groups and in different settings; and influencing a range of outcomes, i.e. it is a complex intervention [37,38].

### Components of *Healthy Ageing*

Five intervention components were incorporated: a mass media campaign, information meetings for interested local elderly people, psychosocial group courses for persons with mental health problems or chronic diseases, social activation by the community-based *Neighbours Connected* intervention [39], and training of intermediaries (homecare nurses, municipal advisors, and volunteers). The general elderly population and persons in their social environment were approached by means of a mass media campaign, including a stand at the municipal information fair in 2008 and 2009, a monthly article in the local newspaper, the distribution of a municipal information booklet, posters with an appealing slogan, and brochures. The information meetings were hosted and advertised by elderly associations and intended for their members in the first place. During the meeting, 10 tips about healthy ageing were discussed [40]. The psychosocial courses were directed at elderly people with mild depressive symptoms and chronic diseases, and focused on the development of coping and communication skills with regard to, e.g., stress situations, personal energy balance, and assertiveness. The group courses, consisting of eight to 10 meetings, were based on the principles of life history memory, shown to be effective for small-size depression reduction [41-43]. Participants were recruited by advertisements in the newspaper, leaflets in the waiting room of general practitioners (GP), and GP referral. *Neighbours Connected* was a newly developed sub-project of *Healthy Ageing* in which citizens were stimulated, and financially and practically supported, to organise a social activity [39]. Activities were organised in the neighbourhood, and organisers personally invited socially inactive neighbours to join the activity. All in all, the intervention components directed at the primary target group mainly targeted network development and focused to a lesser extent on coping with elderly persons’ feelings of loneliness. Finally, for the intermediaries, workshops, Round Table meetings, and newsletters were developed to improve recognition of loneliness symptoms. More details about these intervention activities can be found elsewhere [44,45].

### Aims

Altogether, *Healthy Ageing* aimed to reduce the average loneliness score among non-institutionalised elderly people aged 65 years or over by 10% in two years (September 2008 until September 2010). From a public health perspective, the project was directed at all non-institutionalised older residents and persons in their surroundings. *Healthy Ageing* defined two sub-objectives: 1) to reduce loneliness in the high-risk groups (physical limitations, low income, recent widowhood, mild mental disabilities); and 2) to create more awareness about the existence of loneliness in the general population. At the start, the intervention activities were intended to follow a process of growth during the two-year project period, by mobilising stakeholders and obtaining political commitment. Therefore, the evaluation plan included the evaluation of the individual intervention components as well as of the overall complex intervention in order to be able to detect results at different levels. The aim of this paper is to study the effects of the loneliness intervention *Healthy Ageing* in relation to the initial outcome, loneliness literacy, long-term outcome, social support, and overall goal, loneliness, after two years.

## Methods

### Study design

**Figure 1:**
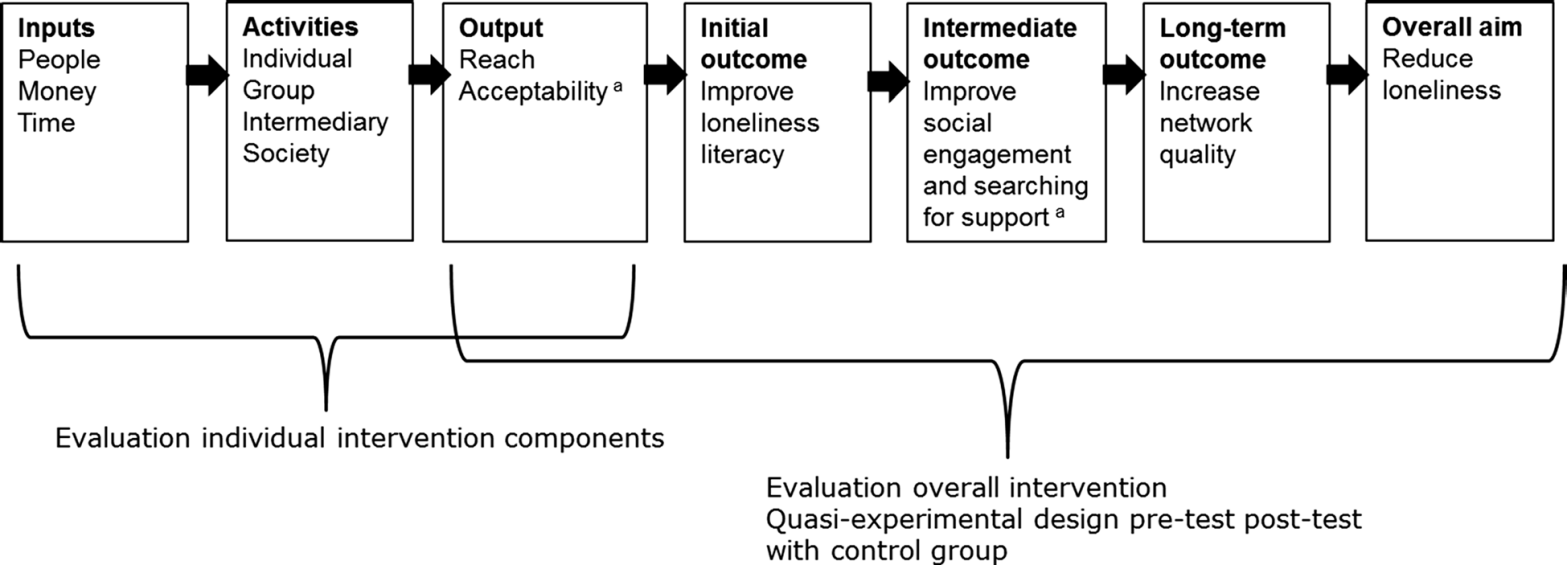
**Logic model of *Healthy Ageing*. **^a^ Not included in this study.

The evaluation of *Healthy Ageing* consisted of two parts, namely, the evaluation of the overall intervention and that of the individual intervention components (Figure 1). To evaluate the overall effect of *Healthy Ageing*, a quasi-experimental pre-test post-test study design was used. A control community (Ermelo) was selected with characteristics comparable to the intervention community (Epe). In the control community, the usual municipal health and welfare services and social activities were offered. Data were collected by means of a self-administered written questionnaire over an 11-week period from mid-August to the end of October in 2008 and 2010, respectively. To evaluate the contribution of the individual intervention components to the overall effect, different small scale studies took place. Data about the reach and acceptability of the individual intervention components were collected by means of registries of project team members, short evaluation forms after activities, and interviews [45]. The current study focuses on the output indicator, reach.

### Study participants and data collection

**Figure 2:**
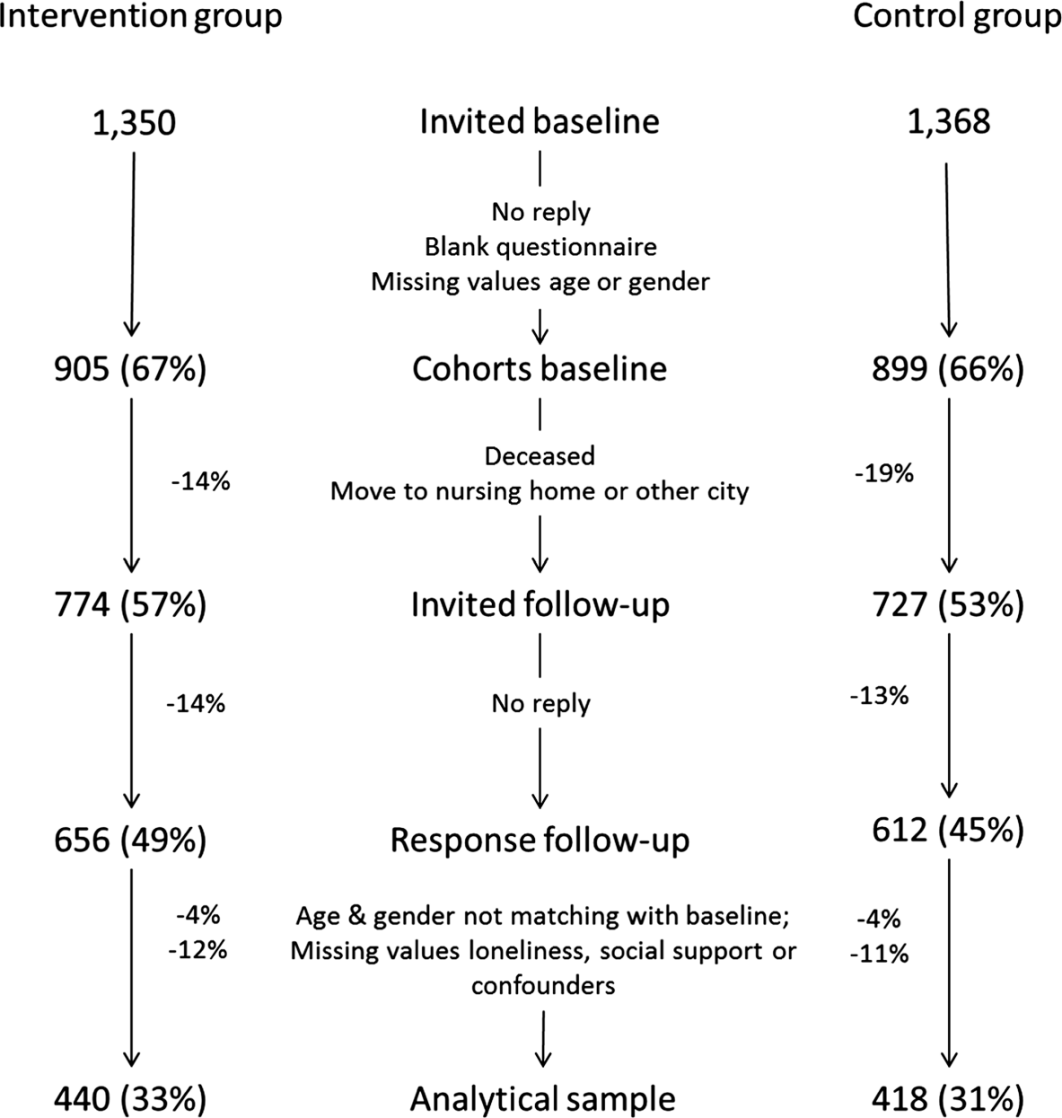
Flow chart of participants and response rates for questionnaires at baseline and follow-up measurements.

From both the intervention and control community, a random sample of 1,350 non-institutionalised elderly people aged 65 years and over was selected from the municipal administration. People aged 75 years or over were oversampled to constitute half of the study population [44]. At baseline, an invitation letter was sent to the home address of the selected inhabitants. If necessary, a reminder was sent after four weeks. A second reminder was sent after seven weeks and included another written copy of the questionnaire. Complete baseline data were obtained from 905 (67%) and 899 (66%) participants in the intervention and the control community, respectively. Fourteen per cent and 19%, respectively, of these study participants were not accessible for the follow-up measurement in 2010 because they had moved to another city or to a nursing home, or were deceased. During the follow-up measurement, the same invitation and reminding procedure was followed. Approximately 14% of the participants invited at follow-up did not respond. Accordingly, persons with differences in reported gender and/or year of birth between two measurement points (4%) or missing values for the main outcome variables and confounders (12% and 11%, respectively) were excluded from the analyses, in total 16% and 14%, respectively. This resulted in a final two-year follow-up analytical sample of 440 (33%) and 418 (31%) participants in the intervention and the control group, respectively (see Figure 2).

The Medical Research Ethics Committee of Wageningen University confirmed that the study, did not fall within the remit of the Dutch ‘Medical Research Involving Human Subjects Act’. The use of personal data in this study is in compliance with the Dutch Personal Data Protection Act and the Municipal Database Act, and has been registered with the Dutch Data Protection Authority (number 1440826).

### Logic Model

A logic model has been developed to guide the evaluation planning. This is described in an earlier paper of the authors [44]. The model focuses on the causal chain between intervention activities and outcomes at the level of the primary target group, i.e. elderly people. In this model, reduction of the prevalence of loneliness is placed as the overall goal. Improvement of the network quality is defined as an early marker for loneliness reduction and the long-term outcome of the intervention. Social support is choses as indicator for network quality. Accordingly, social engagement and searching for professional or informal support were indicated as behavioral outcomes necessary to improve the network quality, and were included as mid-term outcomes. Furthermore, after the example of the outcome model for health promotion of Nutbeam (1998) [46], behavioral determinants for social engagement and searching for support were systematically identified, resulting in the loneliness literacy constructs self-efficacy, perceived social support, motivation and subjective norm [47-50]. Changes in the literacy constructs are expected if sufficient outputs are delivered in terms of reach, dose received, and acceptability. Based on this model, appropriate indicators and research methods have been selected to measure these outcomes (Figure 1).

### Measurements

The questionnaire included the key indicators of the logic model. Furthermore, socio-demographic and health-related determinants were included to describe the study population and to control for confounding.

#### Intervention output-reach

Reach was assessed by two different means. First, ‘dose delivered’ or ‘theoretical exposure’ was assessed by recording the implementation and delivery of, and attendance at, the intervention activities (recorded delivery and recorded reach). Secondly, ‘dose received’ was assessed in the post-test among study participants in the intervention group (recalled reach). In the post-test questionnaire, familiarity with the stand at the information fair, posters with slogan, newspaper articles, information meetings, psychosocial courses, and *Neighbours Connected* was individually questioned. Familiarity with any of the intervention components was calculated by summing the individual seven items. Furthermore, participation in, or organisation of, a *Neighbours Connected* activity and attendance at an information meeting were questioned (recalled participation).

#### Initial outcome-loneliness literacy

The Loneliness Literacy Scale was developed and validated to measure determinants relating to the behaviours ‘becoming or staying socially active’ and ‘searching for support’ [49]. This 22-item scale was included in the post-test measurement and consists of 22 items divided over four subscales, namely, motivation (referring to awareness about, expected outcomes of, and intention to use health and welfare services), self-efficacy (referring to perceived ability to interact socially), perceived social support (referring to previously experienced social support and the motivation to comply with the opinion of important others), and subjective norm (referring to respondents’ personal opinion and the perceived opinion of others with regard to participating in social activities). Responses to the questions were formulated on a 5-point Likert-scale ranging from ‘(fully) agree’ to ‘(fully) disagree’. Sum scores for each subscale were calculated by dividing the totalled scores on the filled out items by the total number of items per subscale, allowing a maximum of one missing value for each subscale. A higher score on each subscale represents a less favourable literacy level.

#### Long-term outcome-social support

Social support was measured using the short version of the Social Support List-Interactions (SSL12-I) by which the extent of received social support by means of social interactions with members of the primary social network was assessed [51-53]. The SSL12-I consists of 12 items, which can be divided equally over three subscales, namely, everyday support (referring to social companionship and daily emotional support), support in problem situations (referring to instrumental support, informative support, and emotional support in times of trouble), and esteem support (referring to support resulting in self-esteem and approval). Responses to these questions were formulated on a 4-point Likert-scale indicating ‘seldom or never’, ‘now and then’, ‘regularly’, and ‘very often’. The subscale scores ranged from 4 to 16 and the score for total social support ranged from 12 to 48. A higher score indicates that more support is experienced. The psychometric properties of the SSL12-I were found to be rather satisfactory in a sample of the Groningen Longitudinal Aging Study with persons aged 57 years and over [51].

#### Overall goal–loneliness

Loneliness was measured using the De Jong Gierveld loneliness scale [54,55]. This scale is composed of 11 questions, of which five are positively and six negatively formulated. Three answer categories were provided (yes, more or less, no). For the positive items, ‘no’ and ‘more or less’ answers were an indication of loneliness, whereas for the negative items ‘yes’ and ‘more or less’ were an indication of loneliness. A higher score represents an increase in severity of loneliness. A score of 0 to 2 corresponds to no loneliness, 3 to 8 to moderate loneliness, 9 to 10 to severe loneliness, and 11 to very severe loneliness. The De Jong Gierveld loneliness scale permits one missing value per respondent to which a score of 0 is given [54-56]. Validity and reliability of the scale [54,55,57] are reported to be satisfactory.

### Background variables: socio-demographic and health characteristics

The socio-demographic characteristics age, sex, marital status, education level, managing on income, and social engagement were included in the study. Marital status was categorised into married or living together, divorced or living separately, widowed, and never married or never lived together; education into illiterate or primary school, lower vocational education, intermediate vocational education, and higher vocational education or university; and having difficulties with managing on income was classified as ‘having major or moderate difficulties’ or ‘having no difficulties’. Doing voluntary work and church attendance were included as proxy for social engagement. Voluntary work was classified as almost daily or weekly, or less frequent; regular church attendance as yes or no.

Suffering from chronic diseases was derived from a list of 13 chronic diseases and categorised into ‘suffering from one or more diseases’ or ‘no diseases reported’, as diagnosed by a physician during the past 12 months. Functional status was assessed using the Hierarchical Abilities of Daily Living (ADL) [58], consisting of 13 activities of daily living categorised in three domains, namely, basic activities of daily life (BADL), mobility activities of daily life (MADL), instrumental activities of daily life (IADL). Persons were assessed for each domain on the basis of being able to perform activities without difficulty or with minor difficulty, versus able to perform with major difficulty or not able to perform the activity without help from others. The Dutch version of the Mental Health Inventory (MHI-5), consisting of two positively and three negatively formulated questions, was included in the questionnaire to assess general mental health. MHI-5 scores ranged from 0 (poor) to 100 (excellent). Good mental health was determined as having a score above 60 [59-63]. Self-perceived health was assessed using the question: ‘How would you classify your health in general?’, using a 5-point scale ranging from excellent to poor. Good self-perceived health was defined as having good, very good, or excellent health [64].

### Study size and data analysis

Descriptive statistics about the delivered dose of the intervention components were derived from records. These records were converted to a population coverage based on the total number of (elderly) inhabitants within the intervention municipality. Furthermore, familiarity with the intervention activities was derived from the post-test and prevalence estimated based on the number of study participants in the intervention group.

The study size for the quasi-experimental study was based on the intended 10% reduction in loneliness, i.e. from a mean score of 2.6 to 2.4 on the De Jong Gierveld loneliness scale [54,55]; 930 individuals with complete data were needed in both the intervention and the control group (α = 0.05;1-β = 0.80). The sample size was raised to 1,350 participants in both groups to compensate for an anticipated response rate of 70% [7]. Background characteristics of the two study populations and their mean scores for loneliness and the social support subscales at baseline were compared using the chi-square tests (categorical variables) and independent samples T-tests (continuous variables). To enable analysis of change in loneliness and the social support subscales, change scores were calculated by subtracting baseline scores from follow-up scores (2010 minus 2008), positive values indicating an increase in either loneliness or social support.

To evaluate the effect of the intervention, linear regression models were constructed with the change scores as dependent variable, with an indicator variable for the intervention (intervention community versus control community) as the effect measure. Adjustment was done for age and gender, followed by additional adjustment for mental health and church attendance (final model). For loneliness literacy, similar analyses were conducted albeit without subtraction of baseline scores as these were not available. In addition to the effect measures obtained from the regression models, effect sizes were expressed in percentages, relative to the baseline scores for loneliness and social support in the intervention community, or relative to the follow-up score for loneliness literacy. Finally, similar analyses were conducted within the intervention community to compare participants who were familiar with one or more of the intervention activities with those who were not. These analyses were restricted to the intervention community, and adjusted additionally for baseline values of marital status, education, church attendance, and regular volunteer work (final model). All analyses were conducted using the software IBM SPSS Statistics 19.

## Results

Mean age was 74 years in both groups and on average 70% of the participants were married. Baseline scores for loneliness, total social support, and the social support subscales did not differ significantly between the intervention and the control group. There were more participants with poor mental health in the intervention than in the control group (14% versus 8%, p <0.01), whereas church attendance was lower in the intervention group (43% versus 60%, p <0.01). For the other determinants, the differences were not statistically significant (Table 1).

**Table 1:** Socio-demographic and health characteristics intervention and control group at baseline

		**Intervention**	**Control**
		**(n = 440)**^**a**^	**(n = 418)**^**a**^
**Age (%)**	**65–75 years**	61	59
	**75+ years**	39	41
	**Mean (sd) age (years)**	73.6 (5.9)	73.8 (6.4)
**Gender (%)**	**Male**	44	47
**Marital status (%)**	**Married/living together**	71	69
	**Never married/never lived together**	3	2
	**Divorced/separated**	4	4
	**Widowed**	23	25
**Education (%)**	**Illiterate/primary education**	18	18
	**Low**	51	45
	**Intermediate**	14	16
	**High**	18	22
**Difficulties managing on income (%)**		12	10
**One or more chronic diseases (%)**		73	79
**Mentally unhealthy (%)**		14*	8*
**Self-perceived health poor (%)**		22	18
**Difficulty BADL**^**b **^**(%)**		4	4
**Difficulty MADL**^**b **^**(%)**		17	16
**Difficulty IADL**^**b **^**(%)**		33	34
**Loneliness (%)**	**Not lonely (0-2)**	51	55
	**Mildly lonely (3-8)**	41	39
	**Severely lonely (9-10)**	7	5
	**Very severely lonely (11)**	2	2
	**Mean (sd) score loneliness**	3.18 (3.13)	2.89 (2.89)
**Social support**	**Total social support-Mean (sd)**	28.31 (6.09)	28.62 (5.73)
	**Everyday social support**	10.37 (2.10)	10.32 (1.86)
	**Support in problem situations**	8.74 (2.54)	8.79 (2.45)
	**Esteem support**	9.21 (2.38)	9.50 (2.29)
**Doing voluntary work frequently (%)**		16	21
**Church attendance (%)**		43*	60*

*Significant difference between intervention and control group (Chi-square or *t*-test; p < 0.05).

^a^Percentages exceed 100% due to rounding off; for individual variables 0.2 to 4% of data may be missing.

^b^Difficulties in activities of daily living (ADL) are hierarchical; persons with difficulties in BADL are likely to be also included in MADL and IADL.

### Intervention output-reach

Table 2 presents the recorded delivery, recorded reach, recalled reach, and recalled participation. With regard to the mass media campaign, the project team of *Healthy Ageing* was present at the municipal information fair in two successive years. Furthermore, each month (except holiday periods) an article was published in the local newspaper, and posters with an appealing slogan were distributed in the municipality in months 2, 14, and 18 of the two-year period. In addition, 11 information meetings, 10 activities of *Neighbours Connected*, and two psychosocial courses were organised and attended by respectively 350, 220, and eight residents, The population coverage for the individual intervention components was estimated between 0.1 and 6%.

**Table 2:** **Dose delivered and dose received of intervention components of *Healthy Ageing *** **directed at the primary target group in the period 2008-2010**

**Activity**	**Dose delivered-Records**		**Dose received-Post-test**	
	**Delivery**	**Recorded reach (population coverage)**	**Recalled reach: study population’s familiarity with activities (n = 440)**	**Recalled participation**
**Information fair**	Yearly one day	± 80 visitors each year	40 (9%)	n/a
	Twice in total	± 160 in total (0.5%)^a^		
**Newspaper article**	Monthly publication	Distributed door-to-door, no recorded data available	87 (20%)	n/a
	19 in total			
**Posters with slogan**	Three mailings to 190 addresses	Not evaluated	8 (2%)	n/a
	(e.g. municipal offices, GPs, physiotherapists, housing agencies for the elderly, welfare organisations, etc.)			
	Print number 100			
**Information meeting**	11 workshops	11-100 participants per workshop, on average 33 participants per meeting	84 (19%)	11 (3%)
		±350 in total (6%)^b^		
**Course *Life Stories***	1 course (8 meetings)	4 participants (0.1%)^b^	35 (8%)	1 (0.2%)
**Course *Living with a Chronic Disease***	1 course (10 meetings)	4 participants (0.1%)^b^	39 (9%)	0
***Neighbours Connected***	10 activities	6-50 participants per activity	48 (11%)	8 (2%)
		± 220 participants in total (4%)^b^		
**Familiar with one or more activities**			**172 (39%)**	

^a^Estimated percentage of all citizens in the intervention community (± 33,000 inhabitants).

^b^Estimated percentages of total target population (± 6,200 persons aged 65 years and over).

Thirty-nine per cent of the intervention group participants stated that they were familiar with one or more of the abovementioned intervention components. The newspaper articles (20%) and information meetings (19%) were the best known intervention components; 10% of the participants were familiar with both.

### Initial outcome-loneliness literacy

At follow-up the intervention group scored significantly better on the loneliness literacy subscales motivation (4%), perceived social support (8%) and subjective norm (12%) than the control group (see Table 3, final model and relative effect size). In line, participants who were familiar with *Healthy Ageing* scored better on the loneliness literacy subscale motivation (6%) and subjective norm (9%) than participants who were not familiar with the intervention, however these differences were borderline significant.

**Table 3:** **Effect evaluation of initial outcomes on loneliness literacy: mean (sd) follow-up scores loneliness literacy and regression coefficients for the comparison of the intervention (n = 372) versus the control group (n = 339) and participants who were familiar (n = 152) versus participants who were not familiar (n = 220) with *Healthy Ageing***

**Initial outcome loneliness literacy**	**Mean (sd) follow-up**^**a**^		**Effect estimates**			
			**Crude effect**^**b**^	**Age- and gender adjusted**^**c **^**(p-value)**	**Final model**^**d **^**(p-value)**	**Relative effect size**^**e **^**% (95% CI)**
**Intervention/control**	**Intervention**	**Control**				
LL motivation	2.98 (0.74)	3.07 (0.77)	−0.09	−0.09 (0.12)	−0.13 (0.02)*	−4.4 (−8.3; −0.7)
LL self-efficacy	1.93 (0.76)	1.86 (0.81)	0.08	0.07 (0.20)	−0.01 (0.87)	−0.5 (−6.0; 15.1)
LL social support	2.07 (0.77)	2.17 (0.80)	−0.10	−0.11 (0.07)	−0.17 (0.01)*	−8.2 (−13.6; −2.4)
LL subjective norm	2.44 (1.00)	2.65 (1.00)	−0.21*	−0.20 (0.01)*	−0.28 (0.00)*	−11.5 (−17.4; −5.4)
**Intervention only**	**Familiar**	**Not familiar**				
LL motivation	2.84 (0.64)	3.07 (0.79)	−0.22*	−0.20 (0.01)*	−0.16 (0.06)	−5.6 (−11.5; 0.14)
LL self-efficacy	1.86 (0.68)	1.97 (0.83)	−0.11	−0.17 (0.04)*	−0.06 (0.46)	−3.2 (−12.2; 5.6)
LL social support	2.02 (0.77)	2.09 (0.77)	−0.07	−0.07 (0.38)	−0.06 (0.51)	−3.0 (−11.8; 5.9)
LL subjective norm	2.32 (0.97)	2.55 (1.00)	−0.23*	−0.23 (0.04)*	−0.20 (0.08)	−8.6 (−18.4; 1.2)

*significant at p < 0.05.

^a^Lower loneliness literacy scores are more favourable.

^b^Difference in mean score at follow-up between intervention group and control group; or between participants who were or were not familiar with the intervention components.

^c^Multivariate model for the comparison intervention versus control, and for the comparison familiar versus not familiar, adjusted for age and gender.

^d^Multivariate model for the comparison intervention versus control additionally included church attendance and mental health. The model comparing familiar versus not familiar additionally included marital status, education, church attendance, and doing voluntary work.

^e^Effect measure obtained from final model relative to the follow-up score for loneliness literacy in the intervention community.

### Long-term outcome-social support

Total social support, everyday support and social support in problem situations significantly increased in the intervention and the control group after two years. Esteem support only increased in the intervention group. However, no differences were found between the changes in the intervention and control group for total social support and the social support subscales (Table 4). Similarly, no significant differences were found for changes in social support between participants within the intervention group who were or were not familiar with the intervention activities.

**Table 4:** **Effect evaluation of intermediate and long-term outcomes: mean change (sd) scores social support and loneliness and regression coefficients for the comparison of the intervention (n = 440) versus the control group (n = 418) and participants who were familiar (n = 172) versus participants who are not familiar (n = 268) with *Healthy Ageing***

**Intermediate and long-term outcomes**	**Mean change (SD) in comparison groups**		**Effect estimates**			
			**Crude effect**^**a**^	**Age-and gender adjusted**^**b **^**(p-value)**	**Final model**^**c **^**(p-value)**	**Relative effect size**^**d **^**% (95% CI)**
**Intervention/control**	**Intervention**	**Control**				
Total social support	1.18 (5.10)	1.00 (5.61)	0.18	0.18 (0.63)	0.20 (0.59)	0.71 (−1.9; 3.3)
Everyday social support	0.16 (1.73)	0.32 (1.75)	−0.16	−0.17 (0.16)	−0.14 (0.26)	−1.4 (−3.6; 1.0)
Support in problem situations	0.67 (2.49)	0.53 (2.69)	0.14	0.14 (0.42)	0.13 (0.46)	1.5 (−2.5; 5.6)
Esteem support	0.34 (2.18)	0.15 (2.25)	0.20	0.20 (0.20)	0.20 (0.20)	2.2 (−1.1; 5.5)
Loneliness	0.05 (2.43)	0.11 (2.43)	−0.07	−0.05 (0.75)	−0.07 (0.67)	−2.2 (−12,0; 7.7)
**Intervention only**	**Familiar**	**Not familiar**				
Total social support	1.18 (4.44)	1.17 (5.48)	0.01	−0.05 (0.93)	−0.07 (0.90)	−0.25 (−4.0; 3.5)
Everyday social support	0.13 (1.63)	0.18 (1.80)	0.05	−0.08 (0.65)	−0.02 (0.91)	−0.19 (−3.8; 3.3)
Support in problem situations	0.75 (2.14)	0.61 (2.69)	0.14	0.14 (0.57)	0.05 (0.85)	0.58 (−5.4; 6.6)
Esteem support	0.29 (2.22)	0.38 (2.16)	0.08	−0.12 (0.58)	−0.12 (0.60)	−1.29 (−6.2; 0.33)
Loneliness	0.24 (2.49)	0.08 (2.38)	−0.33	0.34 (0.18)	0.23 (0.39)	8.0 (−9.9; 25.6)

*significant at p < 0.05.

^a^Difference between mean change in intervention as compared to control group; or difference between mean change among participants who were familiar as compared to not familiar with *Healthy Ageing*.

^b^Multivariate model for the comparison intervention versus control, and for the comparison familiar versus not familiar, adjusted for age and gender.

^c^Multivariate model for the comparison intervention versus control additionally included church attendance and mental health. The model comparing familiar versus not familiar additionally included marital status, education, church attendance, and doing voluntary work.

^d^Effect measure obtained from final model relative to the baseline score for loneliness and social support in the intervention community.

### Overall goal-loneliness

No significant changes in loneliness could be observed over time in either the intervention or the control group. Accordingly, changes did not differ significantly between the intervention and the control group, relative effect size−2.2% (95% CI−12.2-7.7). Similarly, no significant differences were found for changes in loneliness between participants within the intervention group who were or were not familiar with the intervention activities.

## Discussion

Two years after baseline, we found more favourable scores on the loneliness literacy subscales, motivation, perceived social support, and subjective norm (initial outcomes), in the intervention group as compared to the control group. However, we did not find an effect of the complex intervention *Healthy Ageing* on the long-term outcome, social support, or the overall goal, loneliness.

### Characteristics of *Healthy Ageing* project

*Healthy Ageing* was one of the first community projects targeting loneliness among elderly people in the Netherlands. In close collaboration with local authorities and stakeholders, the local project team developed preventive intervention activities adapted to the local organisational infrastructure around preventive elderly health care. Because of this practice-driven approach, it was initially not explicitly stated how the intervention activities would contribute to the formulated objectives. As a result, communication activities to raise public awareness were dominant in the first months. Along the way evidence from literature and local research was incorporated in the project, introducing a more systematic approach and giving explicit attention to network development. Because of this development of the project, the overall reach and intensity of *Healthy Ageing* after two years were modest. Thus, in retrospect, it can be concluded that changes in loneliness and social support could not yet be expected based on the intervention logic.

### Methodological considerations

The SSL12-I and the De Jong Gierveld loneliness scale are considered as reliable and valid instruments to assess received social support and loneliness, respectively [51-53,56,57,65]. The Loneliness Literacy Scale was developed within the framework of *Healthy Ageing* in order to be context and topic specific. The internal consistency of the subscales appeared to be adequate as Cronbach’s coefficient α exceeded 0.7 [49]. Furthermore, the concurrent validity of the scale, cross-sectionally assessed by the association between loneliness literacy and loneliness, appeared to be acceptable for the subscales, self-efficacy, perceived social support, and subjective norm, in the validation study [49], and this was confirmed in the follow-up data of the current study. Responsiveness, i.e. the ability of the instrument to detect change over time in the construct to be measured [66], has not been formally tested for the three selected scales: the De Jong Gierveld loneliness scale, SSL12-I, and the Loneliness Literacy Scale. However, the De Jong Gierveld loneliness scale is frequently used in evaluation studies and appears to be sensitive enough to assess intervention effects [23,24,28,67].

In this study, a quasi-experimental design, including a pre-test and post-test and a control group, was used, which contributes to the internal validity of the results [68]. We could not randomly assign participants to the intervention activities, but selected a rural community with comparable population characteristics as control. Nevertheless, church attendance and mental health differed and were accounted for in the analysis. Unfortunately, for loneliness literacy change scores could not be calculated because only post-test data were available. Because of the comparability of baseline characteristics of the intervention and the control group and adjustment for relevant covariables, we assume that this has not interfered with the results.

Thus, the intervention group scored significantly more favourable on the loneliness literacy subscales, motivation, perceived social support, and subjective norm after two years compared to the control group, but in this two-year programme these effects did not yet progress to changes in social support and loneliness. Selective response at baseline and follow-up might have influenced the estimated effect of the intervention. However, drop-out percentage in the intervention and control group was similar in each step (Figure 2) and characteristics of the drop-outs were highly comparable for both communities. At baseline, respondents’ gender, age, and marital status were comparable with the source population [69]. However, persons who dropped out after baseline were older, more likely to be unmarried, and less educated, in both the intervention and control community. Nevertheless, this resulted in a slightly healthier and less lonely analytical sample. Therefore, the associations found might be either an over-or underestimation of the overall effect in the intervention community as a whole. Within the intervention community, it appeared that those who were familiar with the intervention were already slightly healthier at the pre-test which suggests that healthy people were better reached by the intervention activities. It might be assumed that these healthier elderly persons were better able to incorporate advices in their daily life, resulting in more favourable literacy scores. However, effects of this investment on experienced social support and loneliness will need more time to become measurable. Moreover, among healthier persons there is less room for improvement, resulting in an underestimation of the effect among moderate and severe lonely elderly people.

### Explanation of the observed effect

In order to conclude that the positive effect on the loneliness literacy subscales, motivation, perceived social support, and subjective norm, is a reliable indication of the effect of the intervention, three criteria must be assessed: 1) the strength of the relationships between the intervention and the literacy outcomes; 2) the strength of the theoretical model, i.e. the association between loneliness literacy and loneliness; and 3) the plausibility that the intervention activities could have changed the literacy constructs. Firstly, the effect sizes of the association between the intervention and loneliness literacy subscales, motivation, social support, and subjective norm were meaningful (4.4-11.5%). Furthermore, the effects in persons who were familiar with *Healthy Ageing* pointed in the same direction (3.0-8.6%), albeit of borderline significance. Secondly, the hypothesised logic model between the intervention, loneliness literacy, social support, and loneliness was confirmed as more favourable scores on the loneliness literacy subscales, self-efficacy and social support, were associated with more social support and with less loneliness. However, the subscale, motivation, was not associated with the long-term outcome and overall goal whereas favourable scores on the subscale, subjective norm, were associated with more loneliness and not with social support. Thirdly, the mass media communications and information meetings focused mainly on raising awareness among elderly people and the general population about the importance of social engagement and opportunities to receive professional support or meet other people. The subscale, motivation, included items relating to awareness about these opportunities for support in the municipality. This supports the observed effect on this subscale. The literacy constructs, perceived social support and subjective norm, reflect an individual’s experience about the attitude of important persons in his/her social environment. As argued above, *Healthy Ageing* might have raised awareness among social network members, i.e. the general population in Epe. However, a change in attitude and behaviour among these network members is needed as an additional step before elderly people will experience a difference. Therefore, based on the complexity of the mechanism, it is less likely that the subscales, perceived social support and subjective norm, were changed by *Healthy Ageing*. Finally, skill training and stimulating self-efficacy were mainly embedded in the psychosocial courses. As the reach of these courses was very low (n = 8), an effect on the subscale, self-efficacy, was very unlikely. All in all, regarding the third criterion, it can be concluded that the effect of *Healthy Ageing* on the loneliness literacy subscale, motivation, is plausible, on the subscales, perceived social support and subjective norm, probable, and on the subscale, self-efficacy, unlikely. Whether the effect on motivation is an early indication of effects on the long-term outcome social support and overall goal loneliness, needs further confirmation.

### Comparison with other studies

*Healthy Ageing* distinguishes itself from other loneliness interventions by its community and integrated approach, resulting in a combination of intervention components directed at elderly people themselves and persons in the social environment. Therefore, it is not possible to directly compare our results with other studies. With regard to single interventions, few have proven to be effective in the reduction of loneliness [23,26-28,70], more have shown effects on social indicators such as participation, support satisfaction, and frequency of contacts [27,70]. Initial outcomes such as coping skills and self-confidence are rarely included in the evaluation of loneliness interventions [24,71]. Positive results on loneliness have been attained among specific groups of elderly people with a handicap or chronic disease by an individual internet-at home project [23]. Similarly, evidence of a reduction in depression was found for the psychosocial course included in *Healthy Ageing*[41-43]. Furthermore, social support interventions, such as a friendship enrichment course or discussion groups, aimed at increasing opportunities for social engagement, seem to be promising [23,26,27,72]. However, it has to be noted that the success of loneliness interventions depends largely on the characteristics of the target group, e.g. cause of loneliness and social skills [27], and the local context, e.g. intervention providers and social and physical resources. Within *Healthy Ageing*, the local infrastructure was taken into account, but target group differentiation probably needs further attention to distinguish lonely elderly people, elderly people with an (identifiable) increased risk of loneliness, social network members of elderly people and professionals. These groups have clearly different needs to combat loneliness, requiring different messages and different strategies. Therefore, for *Healthy Ageing* as well as for other community interventions, involvement of representatives of different segments of the local target population and intervention providers during all stages of the intervention is highly relevant and highly recommended [23,26,27]. Finally, more attention should be given to vulnerable elderly people who are at increased risk of becoming isolated and lonely. These people, with the highest needs, are however most difficult to find. In the perspective of the ageing population, using the valuable social capital of healthy elderly people is promising to reach their vulnerable older counterparts.

## Conclusions

To conclude, though the *Healthy Ageing* faces opportunities for improvement, this study did show initial effects on the loneliness literacy subscales, motivation, perceived social support and subjective norm, whereas the effects did not carry forward to the long-term outcome and overall goal, social support and loneliness.

## Abbreviations

GP: General practitioner; SSL12-I: 12-item Social Support List-Interactions; ADL: Abilities of daily living; BADL: Basic activities of daily life; MADL: Mobility activities of daily life; IADL: Instrumental activities of daily life; MHI-5: 5-item mental health inventory.

## Competing interests

The authors declare that they have no competing interests.

## Authors’ contributions

RHdV has taken main responsibility for the study’s data collection, analyses, interpretation of the results, and in writing the first draft and subsequent revision of the manuscript. AH, JH, PvtV, and LdG participated in defining the study design and the editing subsequent revision of the manuscript. All authors read and approved the final manuscript.

## Pre-publication history

The pre-publication history for this paper can be accessed here:

http://www.biomedcentral.com/1471-2458/13/984/prepub
